# Molecular Determinants for Guanine Binding in GTP-Binding Proteins: A Data Mining and Quantum Chemical Study

**DOI:** 10.3390/ijms252212449

**Published:** 2024-11-20

**Authors:** Pawan Bhatta, Xiche Hu

**Affiliations:** Department of Chemistry and Biochemistry, University of Toledo, Toledo, OH 43606, USA; pawan.bhatta@rockets.utoledo.edu

**Keywords:** molecular recognition, quantum mechanics, G protein, GTP-binding protein, π–π stacking interactions, cation–π interaction, hydrogen bond

## Abstract

GTP-binding proteins are essential molecular switches that regulate a wide range of cellular processes. Their function relies on the specific recognition and binding of guanine within their binding pockets. This study aims to elucidate the molecular determinants underlying this recognition. A large-scale data mining of the Protein Data Bank yielded 298 GTP-binding protein complexes, which provided a structural foundation for a systematic analysis of the intermolecular interactions that are responsible for the molecular recognition of guanine in proteins. It was found that multiple modes of non-bonded interactions including hydrogen bonding, cation–π interactions, and π–π stacking interactions are employed by GTP-binding proteins for binding. Subsequently, the strengths of non-bonded interaction energies between guanine and its surrounding protein residues were quantified by means of the double-hybrid DFT method B2PLYP-D3/cc-pVDZ. Hydrogen bonds, particularly those involving the N2 and O6 atoms of guanine, confer specificity to guanine recognition. Cation–π interactions between the guanine ring and basic residues (Lys and Arg) provide significant electrostatic stabilization. π–π stacking interactions with aromatic residues (Phe, Tyr, and Trp) further contribute to the overall binding affinity. This synergistic interplay of multiple interaction modes enables GTP-binding proteins to achieve high specificity and stability in guanine recognition, ultimately underpinning their crucial roles in cellular signaling and regulation. Notably, the NKXD motif, while historically considered crucial for guanine binding in GTP-binding proteins, is not universally required. Our study revealed significant variability in hydrogen bonding patterns, with many proteins lacking the NKXD motif but still effectively binding guanine through alternative arrangements of interacting residues.

## 1. Introduction

GTP-binding proteins, commonly known as G proteins, are critical regulators of a myriad of cellular processes, acting as molecular switches that facilitate signal transduction in response to extracellular stimuli [1,2,3,4]. Two of the most common GTP-binding proteins are heterotrimeric G proteins and small monomeric G proteins [5,6]. Heterotrimeric G proteins consist of three subunits—α, β, and γ—and are integral to signaling pathways mediated by G protein-coupled receptors (GPCRs). They can be further divided into families based on the α subunit, including the Gs family (which activates adenylyl cyclase), the Gi family (which inhibits adenylyl cyclase), the Gq family (which activates phospholipase C), and the G12/13 family (which regulates Rho family GTPases). Small monomeric G proteins, on the other hand, are single polypeptide chains that function independently in various signaling pathways. This group includes the Ras family, known for its role in cell proliferation and survival; the Rho family, which regulates cytoskeletal dynamics; the Rab family, involved in vesicular transport; the Arf family, associated with membrane trafficking; and the Ran family, crucial for nucleocytoplasmic transport. Other GTP-binding proteins include septins, tubulins, dynamins, eukaryotic translation initiation/elongation factors, etc.

Due to the important role of GTP-binding proteins in various cellular processes and diverse signaling transduction networks, the molecular recognition of guanine nucleotides (GTP, GDP, and GMP) in GTP-binding proteins has long been a subject of great interest. The concept of the molecular recognition of ligands in proteins has a historical origin based on Emil Fischer’s “lock-and-key” model and Daniel Koshland’s “induced fit” hypothesis. It is the advent of X-ray crystallography that enabled the ability to visualize the complex three-dimensional structures of GTP-binding proteins and their complexes, thereby elucidating the critical sequence motifs and binding sites integral to molecular recognition [7]. The sequence motif responsible for the binding of guanine to G proteins is G4, i.e., the N/TKXD sequence motif [8,9]. Hereafter, we will adopt the motif nomenclature of [8]. GTP-binding proteins also contain G1, i.e., the GXXXXGK(S/T) sequence motif (also known as the Walker A motif), which is responsible for binding the GTP’s phosphate group. This phosphate group is also present in ATP, and the same G1 sequence motif is responsible for the phosphate group recognition in the ATP-binding protein. The G2, i.e., X(T/S)X, and G3, i.e., DXXG (Walker B motif), sequence motifs are involved in the coordination of Mg^2+^ ions. G5 is the (T/G)(C/S)A sequence motif required to strengthen the guanine base recognition [8]. Multiple studies have been carried out to understand the molecular recognition of ribose sugar [10], a phosphate group, and its associated magnesium ion [11,12,13,14] of GTP and ATP in proteins. In this work, we aim to decipher the molecular determinants for molecular recognition of the guanine base in GTP-binding proteins.

It is now widely accepted that molecular recognition is mediated through non-covalent interactions (also known as non-bonded interactions) such as hydrogen bonding, metal coordination, van der Waals forces (VDW), cation–π interaction, π–π stacking interaction, CH–π interaction, XH–π interaction (X = N, O, S), salt bridge, etc. [15,16,17,18,19,20]. To understand the molecular recognition of guanine, one needs to know the specific non-bonded interactions between guanine and its surrounding residues in proteins.

**Figure 1:**
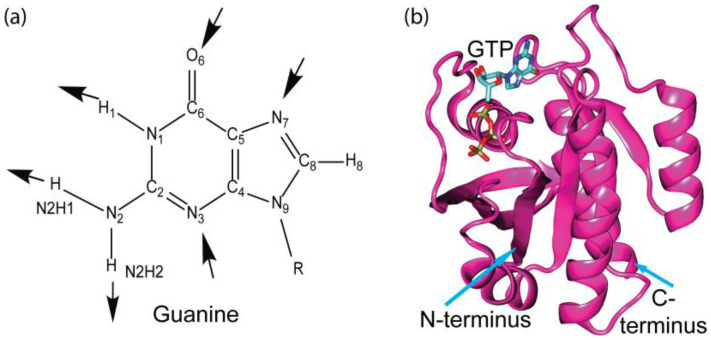
(**a**) The guanine base of the guanine nucleotide (GTP/GDP/GMP), where the symbol R represents ribose and phosphate groups. The inward arrow shows the hydrogen bond acceptor, and the outward arrow shows the hydrogen bond donor. All the atoms are labeled according to the IUPAC naming system. (**b**) Structure of a representative GTP-binding protein: a p21-ras protein bound to GTP (PDB ID:1QRA).

Figure 1 shows the molecular structure of the guanine base, an aromatic motif that features multiple hydrogen bond acceptors and donors, which can form specific hydrogen bonds with the surrounding residues inside the GTP-binding pocket. It has the capacity to form as many as six hydrogen bonds, acting as a donor for three hydrogen bonds at the N1 and N2 positions, and hydrogen bond acceptors at the N3, O6, and N7 positions. This hydrogen bonding capacity of the guanine base is widely accepted as an important non-bonded interaction mode for DNA base-pairing and protein–ligand interactions. There are two more equally important non-bonded interaction modes for guanine–protein binding, i.e., π–π stacking interactions and cation–π interactions. Just as in the case of DNA base stacking, the conjugated π rings of the guanine base can interact with surrounding aromatic residues (Phe, Tyr, and Trp) via π–π stacking interactions. The conjugated π rings of guanine base can also interact with positively charged residues (Lys and Arg) through cation–π interactions. A wealth of information has been accumulated displaying the importance of π–π stacking interactions and cation–π interactions in the formation of biomolecular systems [15,16,20,21,22,23]. Typically, π–π stacking interactions and cation–π interactions are of similar or even greater magnitude than the hydrogen bonding energy, as shown by our ATP binding study [24] and other investigations of cation–π [25,26,27] and π–π stacking interactions [28,29,30] in biological systems. In our analysis of the molecular recognition of the guanine base of GTP reported here, π–π stacking interactions and cation–π interactions are systematically analyzed in addition to hydrogen bonding. Furthermore, contributions of each one of the non-bonded interaction modes (π–π stacking, cation–π interaction, and H-bonding) to binding between the guanine base and protein are quantified by means of high-level quantum chemical calculations. We are interested in determining which types of non-bonded interactions are used by GTP-binding proteins in the recognition of the guanine base and what their relative importance is.

In this study, the molecular determinants responsible for the molecular recognition of the guanine moiety in GTP-binding proteins were deciphered by means of data mining and high-level quantum chemical analysis. A large-scale data mining of the Protein Data Bank was carried out, which resulted in the establishment of a database of 298 nonredundant high-resolution GTP-binding proteins complexed with bound guanine nucleotides. For all these complexes, the modes of the non-bonded interactions between guanine and its surrounding residues were systematically analyzed to decipher the specific interactions responsible for molecular recognition. Furthermore, the contributions of each one of the non-bonded interaction modes (π–π stacking, cation–π interaction, and H-bonding) to binding between the guanine base and protein were quantified by means of high-level quantum chemical calculations.

The remainder of this article is structured as follows. The Results and Discussion section presents the binding environments of the guanine base in 298 guanylate-binding protein complexes, particularly focusing on the dominant hydrogen bonding patterns, the cation–π interactions between guanine bases, and the side chains of positively charged residues lysine and arginine, as well as the π–π stacking interactions between guanine bases and the side chains of aromatic residues such as phenylalanine, tyrosine, and tryptophan. The strengths of non-bonded interactions for the representatives of cation–π and π–π stacking interactions, as calculated using the B2PLYP-D3/cc-pVDZ method [31,32,33], are described. Then, a case study is detailed, illustrating the distribution of the energetic contributions from various modes of non-bonded interactions to the binding of guanine within the context of an entire protein. The biological significance of our findings is discussed at the end of this section. In the Theory and Methods section, we detail the procedures for data mining GTP-binding proteins from the Protein Data Bank (PDB) along with specific details regarding the B2PLYP-D3/cc-pVDZ electronic structure calculations of non-bonded interactions in the guanylate–protein complexes. A brief summary is provided in the Conclusion section.

## 2. Results and Discussion

The data mining analysis resulted in a total of 298 nonredundant high-resolution (2.5 Å or better) GTP-binding proteins complexed with bound guanine nucleotides. Proteins with over 90% sequence identity were excluded to minimize redundancy. Table 1 provides an extensive list of the protein complexes containing bound guanine nucleotides (GMP, GDP, and GTP), along with essential details such as the protein family to which each complex belongs, the nucleotide type, PDB IDs, and the structural resolution. These GTP-binding proteins belong to 57 protein families, which underscores the essential, multifaceted role of GTP in cellular biology. These diverse families cover a broad spectrum of biological functions: from basic cellular processes like protein synthesis (50S ribosome-binding GTPases and EF-Tu) and cell division (FtsZ and septins), to advanced regulatory systems in signaling (the Ras family and ARF family) and immune responses (Interferon-inducible GTPase and AIG1). Many families are directly involved in metabolic pathways (PEPCK and GTP cyclohydrolase I) or are structural and enzymatic scaffolds (MobA-like NTP Transferase and Ferrous Iron Transport), ensuring cellular health and adaptability. Moreover, the diverse spectrum of protein families provides a wide variety of guanine-binding pockets for our molecular recognition study.

Based on the three-dimensional structures, the binding pockets of the guanine bases within their respective target GTP-binding proteins were meticulously analyzed using the Visual Molecular Dynamics (VMD) program to identify residues that engage in non-bonded interactions with each guanine base. Consistent with the physical nature of each type of non-bonded interaction, a cut-off distance of 3.5 Å between the donor and the acceptor was used for hydrogen bonding, and a cut-off distance of 5.6 Å was used for π–π stacking and cation–π interactions. For the former interaction, a slightly longer distance of 3.5 Å, rather than the optimal hydrogen bonding range of 2.8 to 3.2 Å, was adopted to account for the dynamic fluctuations in atomic positions. For the latter interactions, the computed strengths of the solution-phase interaction energies typically diminished beyond 5.6 Å, as indicated by our prior quantum chemical calculations [22]. The non-bonded interactions (hydrogen bonding, π–π stacking, and cation–π interactions) so identified were carefully studied, with details tabulated in Table S1 of the Supplementary Materials. Those non-bonded interactions are described and analyzed below.

### 2.1. Hydrogen Bonding

Table 2 presents a summary of the hydrogen bond patterns between the guanine base and surrounding residues in GTP-binding proteins. The table categorizes the hydrogen bonding interactions based on sequence motifs and associated hydrogen-bonding patterns. An extensively detailed list of interaction mode of hydrogen bonds is given in Table S1.

**Figure 2:**
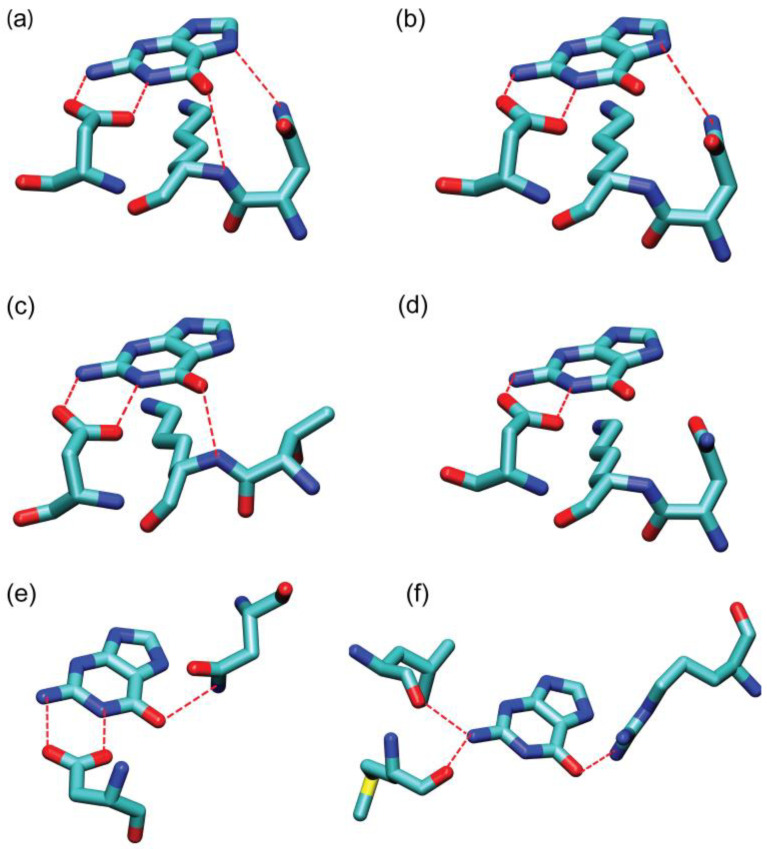
Representative hydrogen bond patterns: (**a**) **N_i_-K_i+1_**-X_i+2_-**D_i+3_** in the p21-ras protein (PDB ID: 1QRA); (**b**) **N_i_**-K_i+1_-X_i+2_-**D_i+3_ **in the Human Ras-like, family 12 protein (PDB ID: 3C5C); (**c**) N_i_-**K_i+1_**-X_i+2_-**D_i+3_** in the human adenylosuccinate synthetase isozyme 2 (PDB ID: 2V40); (**d**) N_i_-K_i+1_-X_i+2_-**D_i+__3_** in the Plasmodium falciparum rab6 protein (PDB ID: 1D5C); (**e**) “D_i_/E_i_ plus” in PnrA from Treponema pallidum (PDB ID: 2FQX); and (**f**) the “Others” pattern in Murray Valley encephalitis virus methyltransferase domain (PDB ID: 2PXA). C, N, O, and S atoms are colored in cyan, blue, red and yellow, respectively.

We identified six distinct hydrogen bonding patterns that are employed by the surrounding residues of the GTP-binding proteins for the molecular recognition of the guanine base. Of these, four are associated with the NKXD motif, while the remaining two patterns lack this motif. For the former, we adopted a hydrogen bonding pattern notation based on the participation of the residues from the NKXD sequence motif in the hydrogen bond interactions. The one-letter residue code is colored red if the residue participates in hydrogen bonding with the guanine base. For clarity, each of the four hydrogen bonding patterns are illustrated with a representative example in Figure 2. Figure 2a depicts the **N_i_-K_i+1_**-X_i+2_-**D_i+3_** pattern, where the side chain of the asparagine (N), the main chain of lysine (K), and the side chain of aspartate (D) form multiple hydrogen bonds with guanine. Figure 2b illustrates the **N_i_**-K_i+1_-X_i+2_-**D_i+3_ **pattern in which the side chain of the asparagine (N) and the side chain of aspartate (D) are involved in hydrogen bonding. Figure 2c shows the N_i_-**K_i+1_**-X_i+2_-**D_i+3_** pattern in which the main-chain amino group from lysine (K) and the side chain of aspartate (D) participate in hydrogen bonds. Figure 2d demonstrates the N_i_-K_i+1_-X_i+2_-**D_i+3_** pattern in which only the side chain of aspartate (D) participates in hydrogen bonding. The first **N_i_-K_i+1_**-X_i+2_-**D_i+3_** and the second **N_i_**-K_i+1_-X_i+2_-**D_i+3_ **pattern both occur most frequently with a probability of 19.1%. The N_i_-**K_i+1_**-X_i+2_-**D_i+3_** pattern appears least frequently with a probability of 6.7%. The N_i_-K_i+1_-X_i+2_-**D_i+3_** pattern was observed in 11.7% of GTP-binding proteins.

Notably, the NKXD motif [9,35]—a fingerprint sequence for guanine-binding sites—emerges as a key player in forming hydrogen bonds with the guanine in these proteins. However, only 56.7% (169 out of 298) of the complexes that bind guanine have the NKXD sequence motif (see Table S1). Additionally, as described above, not all residues within the NKXD motif participate in hydrogen bond interactions.

Remarkably, in the remaining 43.3% of the GTP-binding proteins, the NKXD sequence motif is absent. Within this subset, 20.5% of GTP-binding proteins feature either an aspartate (D) or a glutamate (E) residue that forms hydrogen bonds with the hydrogen from the N1 or N2H1 atoms of the guanine base. We designate this interaction mode as the D/E plus motif, which is illustrated in Figure 2e. The remaining 22.8% of GTP-binding proteins lack a specific conserved sequence motif for guanine recognition, wherein any amino acid residue may form hydrogen bonds with at least one of the N1, N2, or O6 atoms of guanine, either directly or through structured water molecules. An example of the last pattern is given in Figure 2f.

From the perspective of the guanine base, it was found that hydrogen bond donors and acceptors at various positions of the guanine ring have different preferences for hydrogen bond formation. The N1 atom directly donates hydrogen bonds to the surrounding residues, which occurs in 87.3% (260 out of 298) GTP-binding proteins. The N2 atom, on the other hand, has the capacity to directly donate two hydrogens, N2H1 and N2H2, to the surrounding residues. In 89.6% (267 out of 298) of GTP-binding proteins, the N2H1 hydrogen is donated to surrounding residues. In 21.8% (65 out of 298) of GTP-binding proteins, the N2H2 hydrogen is donated to the surrounding residues. In many cases, the N2H2 is also donated to a nearby structured water molecule, which acts as a bridge for hydrogen bonding with amino acid residues in the GTP-binding pocket. It is worth noting that, in many cases, the hydrogen from N1 and N2H1 are donated to aspartate or glutamate to form a double-hydrogen bond (dual-hydrogen bond). Since many GTP-binding proteins have aspartate or glutamate in the GTP-binding pocket, this dual-hydrogen bond mode represents the dominant mode of hydrogen bond interaction. In 27.9% (83 out of 298) of GTP-binding proteins, the O6 atom accepts hydrogen bonds from the main chain amino group of at least one of the residues from the NKXD sequence motif. In 85.6% (255 out of 298) of GTP-binding proteins, O6 accepts an additional hydrogen bond from the main-chain amino group of at least alanine and/or its succeeding residue from the (T/G)(C/S)A sequence motif or any non-conserved residues. The N7 atom accepts a hydrogen bond from the surrounding residue in 55.4% (165 out of 298) of GTP-binding proteins, and the N3 atom accepts a hydrogen bond from the surrounding residues in 6.7% (20 out of 298) of GTP-binding proteins. Interestingly, it was observed that a conserved structured water molecule near the N3 atom donates the hydrogen bond in 41.3% (123 out of 298) of GTP-binding proteins.

Based on the above analysis, it is evident that the most frequent hydrogen bond participating atoms/groups in the guanine base are the N1, N2, and O6 atoms. The N3 and N7 atoms, also present in ATP, are less preferred for hydrogen bonding by proteins that bind guanine.

### 2.2. Cation–π Interaction

**Figure 3:**
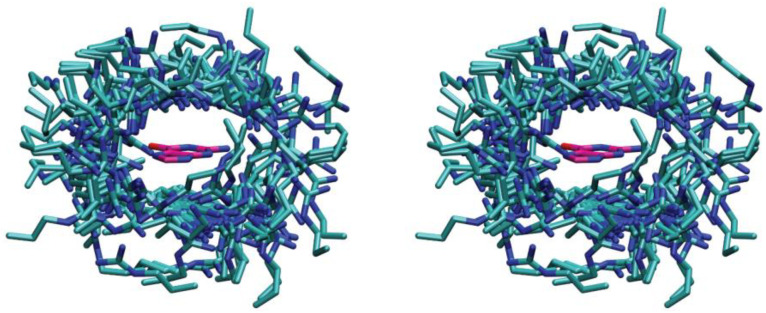
A 3D stereographic drawing of a guanine base surrounded by positively charged residues. All the 258 complexes that contain cation–π interactions are aligned by the superimposition of the guanine base.

Cation–π interactions were systematically examined across all 298 GTP-binding proteins, revealing this interaction as a prevalent non-bonded interaction mode for GTP binding in proteins. In 86.6% of GTP-binding proteins (258 out of 298), at least one cation–π interaction does exist between the guanine base and positively charged side chains of the interacting residues. Furthermore, about 48% of the complexes have more than one positively charged residue interacting with guanine. These complexes are aligned by the superimposition of the guanine base, and Figure 3 shows a 3D stereo drawing of the aligned GTP-protein complexes featuring one or more positively charged residues (lysine and arginine) within 5.6 Å of the guanine base.

**Figure 4:**
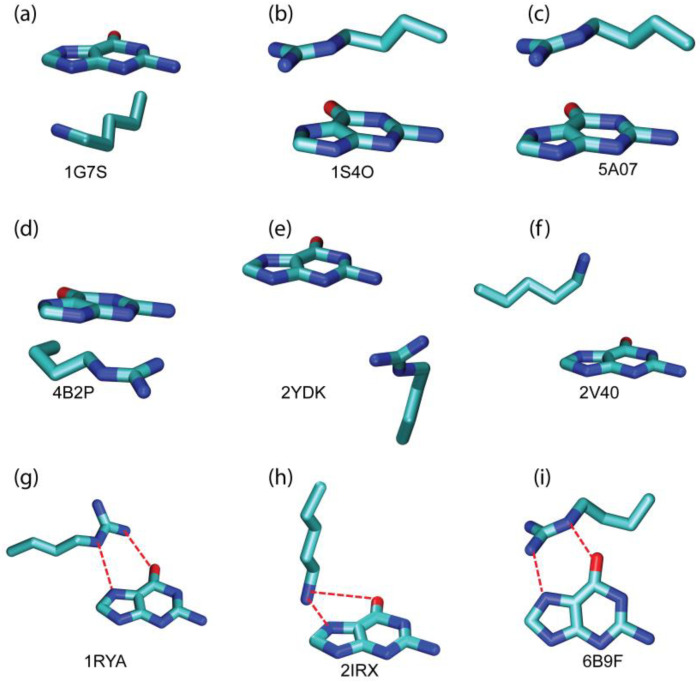
Representative cation–π interactions between the positively charged residues and the guanine ring. PDB IDs for the cation–π interacting motifs are displayed. C, N, and O atoms are colored in cyan, blue, and red, respectively. The red dashed line indicates the coexistence of hydrogen bond interactions.

To quantitatively establish the contribution of cation–π interactions to the binding of guanine with its targeted proteins, the strengths of cation–π interactions between the guanine base and its interacting residues were quantified by means of quantum chemical calculations. For this purpose, 12 distinctive interacting intermolecular pairs between guanine and the positively charged residue were chosen based on Figure 3. They were selected according to two criteria: representation and uniqueness. The representative intermolecular pair is the pair that samples the most abundant regions of Figure 3. The unique intermolecular pair is the one that is uniquely situated in Figure 3 in terms of position and orientation. The three-dimensional structures for 9 of these 12 cation–π interacting pairs are presented in Figure 4. The strengths of the non-bonded interaction energies between guanine and its surrounding aromatic residues were quantified in a pairwise manner using the double-hybrid DFT method at the B2PLYP-D3/cc-pVDZ level of theory (see the Theory and Methods section for details). The resulting pairwise non-bonded interaction energies for the selected cation–π interactions are detailed in Table 3. The magnitudes of cation–π interactions are moderate to strong, ranging from −1.51 to −10.61 kcal/mol. These results indicate the vital role of cation–π interactions in stabilizing guanine binding within GTP-binding proteins.

What are the factors that control the strength of the intermolecular cation–π interaction? This is an important question we want to address below. The intermolecular distance was found to be the predominant factor determining the strength of the non-bonded interaction energy (${\Delta E}_{Int}^{aq}$). As shown in Table 3, as the intermolecular distance increased, the interaction energy decreased. However, at the same time, the extent of overlap between the side chain of positively charged residue and the guanine ring also influenced the intermolecular interaction energy. The representative cation–π intermolecular pairs associated with PDB 1G7S, 1S4O, and 5A07 have the largest interaction energy (see Table 3 and Figure 4). As shown in Figure 4, these cation–π intermolecular pairs have the greatest extent of side chain overlap with the guanine ring. In some unique cation–π intermolecular pairs, a dual mode of non-bonded interactions is seen where cation–π and hydrogen bond interactions both simultaneously exist, e.g., the intermolecular pairs in 1RYA, 2IRX, and 6B9F. The interaction energies are much larger in these cases. This analysis leads us to conclude that the strength of the intermolecular cation–π interaction is dependent upon the combination of three factors, i.e., intermolecular distance, the extent of the side chain overlap of positively charged residues with guanine ring, and the existence of multiple modes of interaction (cation–π and hydrogen bond).

### 2.3. π–π Stacking Interaction

**Figure 5:**
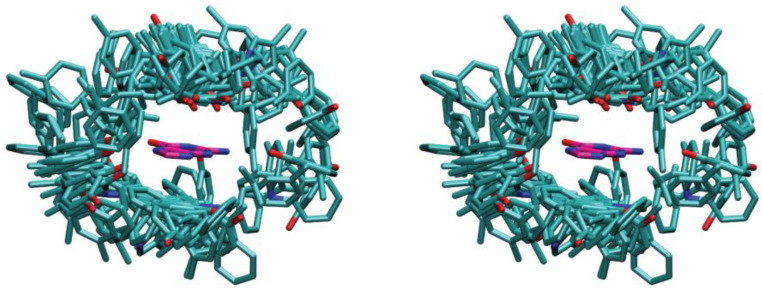
A 3D stereographic drawing of a guanine base surrounded by aromatic residues. All of the 162 complexes that contain π–π stacking interactions are aligned by the superimposition of the guanine base.

The binding pockets of the guanine bases in all 298 GTP-binding proteins were examined to identify the aromatic residues capable of π–π stacking interactions. In 54.4% of the complexes, or 162 out of 298, π–π stacking interaction does exist between the guanine base and aromatic side chains. Figure 5 displays all 162 of the GTP-binding proteins with aromatic residues within 5.6 Å of the guanine base. The aromatic residues Phe, Tyr, and Trp form four primary clusters surrounding the guanine bases: to the left and right, at the top, and at the bottom. The arrangements at the top and bottom are typically categorized as either parallel face-to-face stacking or parallel-displaced stacking, depending on the degree of displacement of the aromatic centers. When aromatic residues perpendicularly approach the π-plane of the guanine base, this configuration is referred to as a “T-shaped” edge-to-face arrangement. The observed distribution pattern of the aromatic residues around the π-plane of the guanine base appears to be optimal, as suggested by modeling studies of the benzene dimer, which is commonly regarded as the standard model for aromatic π–π stacking interactions [36].

**Figure 6:**
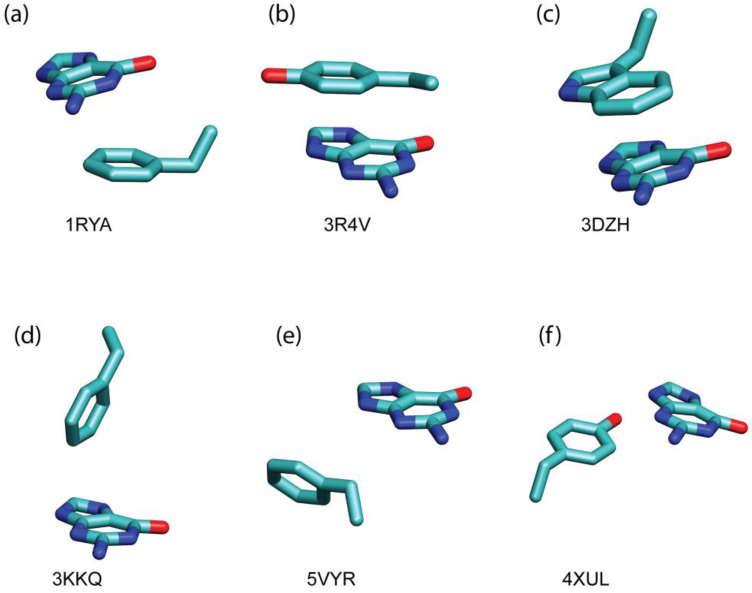
Representative π–π stacking interactions between the aromatic residue and the guanine ring. PDB IDs for the π–π stacking interacting motifs are displayed. C, N, and O atoms are colored in cyan, blue, and red, respectively.

To quantitatively assess the contribution of π–π stacking interactions to the binding of guanine with their target proteins, we employed quantum chemical calculations to evaluate the strength of these interactions between the guanine base and its interacting residues. We selected 14 distinct intermolecular pairs (comprising guanine and aromatic residues) based on the patterns shown in Figure 5. These pairs were chosen according to two criteria: representativeness and uniqueness. A representative pair samples the most prevalent regions in Figure 5, while a unique pair is specifically positioned and oriented within that figure. The three-dimensional structures of 6 of these 14 π–π stacking interacting pairs are illustrated in Figure 6.

The strengths of the non-bonded interaction energies between guanine and its surrounding aromatic residues were quantified in a pairwise manner using the double-hybrid DFT method at the B2PLYP-D3/cc-pVDZ level of theory (see the Theory and Methods section for further details). The resulting pairwise interaction energies for the selected π–π stacking interactions are presented in Table 4.

The π–π stacking interaction energies were found to be low to moderate, ranging from −0.34 to −6.57 kcal/mol. The intermolecular distance is the predominant factor determining the strength of the non-bonded interaction energy (${\Delta E}_{Int}^{aq}$), as can be seen in Table 4 as the intermolecular distance increases the interaction energy decreases. However, at the same time, the angles between two interacting ring planes and the π–π stacking conformations also influence the non-bonded interaction energy. The interaction energy is larger in those cases where the angle between the ring’s planes is nearly zero and the rings are in almost parallel displaced configurations, e.g., the unique π–π stacking intermolecular pair in 1RYA, 3R4V, and 3DZH (see Table 4 and Figure 6). These findings illustrate that π–π stacking interactions, though generally weaker than cation–π interactions, contribute to the stability of the guanine binding in GTP-binding proteins.

### 2.4. Energetic Contribution by Various Modes of Non-Bonded Interactions to the Binding of Guanine in a Representative Complex

The distribution of modes of the non-bonded interactions in the GTP-binding proteins was systematically examined based on their X-ray crystal structures (see Table S1). The objective was to decipher the relative importance of the different modes of non-bonded interactions for the molecular recognition of the guanine base in proteins. Due to space limitation, we chose one GTP-binding protein, i.e., the p21-ras protein (PDB ID: 1QRA), as an illustration. One of the main reasons for the choice of 1QRA is its representativeness; it features the N_i_-K_i+1_-X_i+2_-D_i+3_ hydrogen bond pattern (see Section 2.1 and Table S1). The latter, along with the other three patterns (see above) are associated with the major sequence motif NKXD. On the basis of the 1.6 Å resolution X-ray crystal structure [37] (PDB ID: 1QRA), the binding pocket of the guanine base in the p21-ras protein was thoroughly examined to identify all of the modes of the non-bonded interactions, including hydrogen bonding, salt bridge interactions, π–π stacking interactions, cation–π interactions, CH–π interactions, and XH–π interactions (XH = NH, OH, and SH).

**Figure 7:**
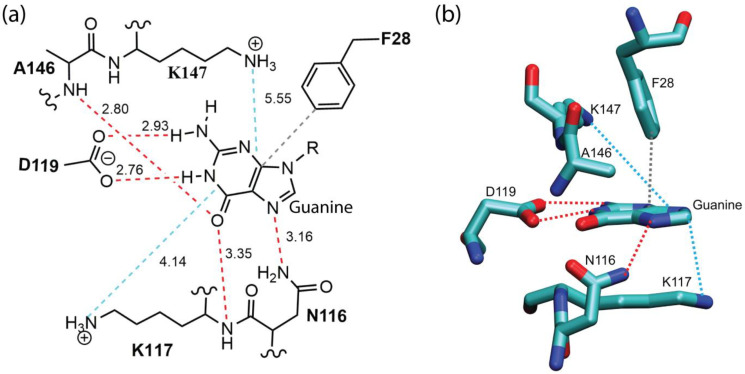
(**a**) A schematic intermolecular interaction map between the guanine and its interacting residues in a GTP-binding protein p21-ras (PDB ID: 1QRA). The interatomic distances (in Å) are indicated along the dashed lines. The red, blue, and gray dashed lines represent hydrogen bond interactions, cation–π interactions, and π–π stacking interactions, respectively. (**b**) The 3D structure of the residues surrounding the guanine. For clarity, only side-chain interactions are shown. The color codes of the dashed lines are the same as in (**a**).

Figure 7 shows the modes of the non-bonded interactions between the guanine base and its interacting residues in the p21-ras protein (PDB ID: 1QRA). The guanine base interacts with its target protein p21-ras via hydrogen bonding, π–π stacking interactions, and cation–π interactions. Either the main chain or the side chain of a residue can form hydrogen bond with guanine. As shown in Figure 7a, there exist multiple hydrogen bonds between the guanine base and the side chains of the Asn116 and Asp119 residues. Interestingly, the carboxyl group of Asp119 forms dual-hydrogen bonds with guanine, where the N2 and N1 atoms of guanine acts as a hydrogen bond donors. In addition, the main chain amino groups of Lys117 and Ala146 donate their hydrogen to the O6 of the guanine ring to form multiple hydrogen bond interactions. The aromatic residue Phe28 is well positioned for π–π stacking interactions with the guanine ring. The ε-amino groups of positively charged Lys117 and Lys147 are involved in cation–π interactions with guanine.

Subsequently, the strengths of the non-bonded interaction energies between guanine and its surrounding protein residues were quantified in a pairwise manner by means of the double-hybrid DFT method B2PLYP-D3/cc-pVDZ (see Theory and Methods for details). The resulting pairwise intermolecular interaction energies between guanine and surrounding residues are listed in Table 5. As shown in the table, the most significant contributor to the interaction energy (${\Delta E}_{Int}^{aq}$) for guanine binding comes from cation–π interactions involving residue Lys117 and Lys147. These interactions account for −18.2 kcal/mol, representing 59.6% of the total binding energy. It is worth noting here that, in addition to the cation–π interactions originating from the positively charged ε-amino groups of lysines, the side-chain alkyl groups of lysine that are parallel to the guanine ring can form multiple CH–π interactions [38] with the guanine ring. The latter enhances the overall strength of the non-bonded interactions involving the lysine residues, as suggested in Ref. [39]. Interestingly, Lys117 also contributes to binding via hydrogen bonds from its main-chain amino group. Hydrogen bonds contribute a total of −9.3 kcal/mol to guanine binding, representing 30.5% of the overall binding energy. This contribution originates from the Asn116 (side chain), Lys117 (main chain), Asp119 (side chain), and Ala146 (main chain) residues. As shown in Table 5, the strengths of those hydrogen bonds vary widely, depending on both the distance and angle. The hydrogen bond energy between Asp119 and guanine was found to be the highest among the interactions analyzed, and it was attributed to its dual interaction mode: both the N1 and N2 groups of guanine donate hydrogen atoms to the oxygen atoms of the Asp119 carboxyl group. In contrast, the hydrogen bond energy between Asn116 and guanine at the N7 position was found to be the weakest. This is due to the extended N–N distance and a suboptimal hydrogen bond angle of 134°, which deviates from linearity and reduces bond strength. Notably, Asn116, Lys117, and Asp119 are part of the NKXD sequence motif (G4 motif), while Ala146 and Lys147 belong to the G5 sequence motif. The π–π stacking interactions between Phe28 and the guanine ring yield an interaction energy of −2.99 kcal/mol.

In summary, the above analysis revealed the energetic hierarchy of the non-bonded interactions in guanine recognition by GTP-binding proteins. Cation–π interactions emerged as the primary source of binding strength, followed by hydrogen bonding for specificity and π–π stacking as an additional stabilizing factor. In particular, the hydrogen bonding interactions between guanine and the side chains of the Asn116 and Asp119 residues, as well as the cation–π interactions between guanine and the positively charged side chains of Lys117 and Lys147, were found to be responsible for the needed specificity and affinity for molecular recognition. In addition, the participation of the main chains of the Lys117 and Ala146 residues in hydrogen bonding interactions with guanine further enhances binding affinity. Furthermore, π–π stacking interactions also meaningfully contribute to guanine binding. These findings are significant as the residues involved in these interactions are derived from the classical NKXD sequence motif and the (T/G)(C/S)A sequence motif (G5 motif). The NKXD motif has evolved as a highly effective binding framework that enables proteins to distinguish guanine from other nucleotides like adenine with remarkable precision. Its specific interactions with guanine’s unique functional groups, combined with a flexible structural arrangement, allow NKXD to achieve high specificity while also supporting diverse binding configurations across protein families.

### 2.5. Biological Significance

The core principle of molecular recognition is the complementarity between a ligand and its receptor, akin to the “lock and key” model, where the receptor serves as the lock and the ligand acts as the key that forms a specific ligand–receptor complex. Over the years, this lock and key model for the molecular recognition of GTP has been explored across various levels of protein structural hierarchy, including sequence motifs, folds, structural motifs, and intermolecular protein–ligand interactions. In 1987, Dever et al. investigated the structural features that define the GTP-binding domain across nine functionally diverse protein families based on primary sequences [40]. It led to the identification of three consensus sequence motives essential for GTP binding: GXXXXGK, DXXG, and NKXD. These elements are spaced 40–80 amino acids apart in most GTP-binding proteins, aiding in recognizing and binding GTP. Since these consensus sequences are conserved among functionally distinct proteins, including elongation factors, the ras protein family, and G proteins, that work suggests a potential application of these motives to screen GTP-binding function from the primary protein sequences of the unknown protein [40]. The subsequent X-ray crystallographical structural determination of the three-dimensional structures of GTP-binding proteins and their complexes confirmed the structural role of the NKXD motif in guanine binding [7,9,41]. Since then, the NKXD motif has been widely viewed as a fingerprint for GTP-binding proteins [9]. In this study, we conducted an analysis of the molecular recognition of the guanine moiety of GTP in the GTP-binding proteins at the level of non-bonded intermolecular interactions. Traditionally, the NKXD sequence is understood as a key motif for guanine binding, where conserved residues directly participate in hydrogen bonding with guanine [9,35,42]. However, this study identifies a surprising level of variability in the hydrogen bonding roles of NKXD residues. Only 56.7% of complexes containing guanine use the NKXD motif for hydrogen bonding, suggesting a broader structural flexibility than previously recognized. Furthermore, 43.3% of guanine-binding proteins lack the NKXD motif entirely, yet they still achieve guanine recognition through alternative hydrogen bonding arrangements. Specifically, proteins without NKXD often utilize aspartate or glutamate in what is described as the “Di/Ei plus” pattern, while others form non-specific hydrogen bonds with a variety of residues. This expanded understanding of the NKXD motif’s variability and the presence of alternative bonding patterns underscores a structural adaptability in guanine recognition, allowing a wider range of proteins to effectively bind guanine despite lacking the classic NKXD sequence. Thus, from the point of view of molecular recognition, this work strongly supports the widely accepted view that non-bonded interactions are the underlying force behind the molecular recognition of a ligand within a protein. Proteins with entirely different folds can adopt analogous recognition schemes characterized by shared protein–ligand interactions.

The hydrogen bonding characteristics of the guanine base are critical for understanding how proteins differentiate between GTP and ATP, and thus merit further discussion. The analysis above indicates that the N2 and O6 atoms in guanine are among the most commonly involved in hydrogen bonding. These specific hydrogen bonds not only stabilize guanine, but also prevent similar binding with adenine as it lacks the N2 and O6 atoms. In contrast, ATP-binding proteins predominantly engage the N1 and N6 atoms of the adenine base for hydrogen bonding [24]. Bear in mind that the N1 atom of guanine is a hydrogen bond donor while that of adenine is an acceptor. Thus, the NKXD motif confers a selective advantage, allowing proteins to differentiate guanine with high fidelity, which is essential for processes where precise nucleotide recognition underpins cellular signaling and function.

We deciphered the molecular determinants involved in the intermolecular recognition of the guanine moiety of GTP by proteins. Our focus is on understanding the types of interactions employed by enzymes for the recognition of the guanine base and their relative importance. In addition to confirming the importance of well-established hydrogen bonding, we found that two additional forms of non-bonded interactions—π–π stacking and cation–π interactions—are also crucial for the guanine binding in proteins. High-level density functional theory (DFT) calculations further support this by demonstrating the significant contributions of hydrogen bonding, π–π stacking, and cation–π interactions to the overall binding affinity of GTP within proteins. It is important to note that previous studies on protein–ligand interactions, particularly those involving GTP, have primarily emphasized hydrogen bonding and hydrophobic interactions [9,40]. However, the data mining and quantum chemical analyses presented here clearly indicate that π–π stacking and cation–π interactions also play critical roles in the binding of the guanine moiety of GTP to proteins.

## 3. Theory and Methods

### 3.1. Data Mining

To establish a database of GTP-binding proteins, a comprehensive data mining of the Protein Data Bank was performed (https://www.rcsb.org). We focused on high-resolution crystal structures (2.5 Å or better) and excluded proteins with over 90% sequence identity to minimize redundancy. Only structures bound to GTP, GDP, or GMP were considered. This resulted in 298 distinct high-resolution crystal structures of GTP-binding protein complexes.

### 3.2. Analysis of Interaction Modes

First, the crystal structures of all 298 GTP-binding protein complexes were aligned by the superimposition of the guanine base using the Visual Molecular Dynamics (VMD) program [43]. Then, the non-bonded interaction modes, i.e., hydrogen bond, cation–π interaction, and π–π stacking interactions between each guanine base and its surrounding residues in each of the 298 complexes, were systematically analyzed to decipher the specific interactions responsible for molecular recognition. A database of such interaction modes was established, with complete details listed in Table S1.

### 3.3. Quantification of Intermolecular Interaction Energy

The framework for the ligand–protein complex formation in solution is illustrated by the following scheme:

(1) $$P(\mathrm{aq})\;\;\;\;\;\;\;\;+\;\;\;\;\;\;\;\;L(\mathrm{aq})\;\;\;\;\overset{{\Delta E}_{int\;}^{aq}}{\to }\;\;\;\;PL(aq)\;{\Delta G}_{P}^{sol}↑\;{\;\;\;\;\;\;\;\;\;\;\;\;\Delta G}_{L}^{sol}↑\;\;\;\;\;\;\;\;\;\;\;\;\;\;\;\;↑{\Delta G}_{PL}^{sol}.\;P(g)\;\;\;\;\;\;\;\;+\;\;\;\;\;\;\;\;L(g)\;\;\;\;\;\;\;\;\;\underset{{\Delta E}_{int\;}^{g}}{\to }\;PL(g)$$

This scheme underpins our analysis of guanine–protein binding affinities. Similar schemes have been used for the solution-phase binding affinity calculations of ligand–protein complexes in previous works [20,22].

Proteins and ligands lose part of their solvation shell upon binding, incurring dehydration energy. The binding energy in solution is thus evaluated via gas-phase intermolecular interaction energies ${\Delta E}_{Int}^{gas}$ corrected for dehydration energy ${\Delta E}_{Deh}$:

(2) $${\Delta E}_{Int}^{aq}={\Delta E}_{Int}^{gas}+{\Delta E}_{Deh}$$

Gas-phase interaction energies were calculated using the supermolecular approach. In the supermolecular approach, the gas-phase energy of the interaction between molecules P and L is defined as the difference between the energy of the interacting dimer $E_{PL}$ and the sum of the energies of monomers $E_{P}$ and $E_{L}$.

(3) $${\Delta E}_{Int}^{gas}=E_{PL}–(E_{P}+E_{L}).$$

The intermolecular interaction energy calculations were performed using Gaussian 09 software by means of the B2PLYP double-hybrid functional [31,44] with Grimme’s D3BJ dispersion correction [32] in conjunction with the cc-pVDZ basis set [33] (B2PLYP-D3/cc-pVDZ). The basis set superimposition error was corrected by the Boys and Bernardi Counter Poise Method [45].

Dehydration energy is defined as the difference of free energy of solvation:

(4) $${\Delta E}_{Deh}={\Delta G}_{PL}^{Sol}-{\Delta G}_{P}^{Sol}-{\Delta G}_{L}^{Sol}$$

Due to high costs of explicit solvent simulations, the free energy of solvation was computed by applying the SM5.42R solvation continuum model by Cramer and Truhlar [46], as implemented in GAMESS [47].

## 4. Conclusions

In this study, we deciphered the molecular determinants essential for guanine recognition in GTP-binding proteins using a multifaceted approach, encompassing large-scale data mining, in-depth analysis of interaction modes, and rigorous quantum chemical calculations. It was found that multiple modes of non-bonded interactions are employed by GTP-binding proteins to achieve molecular recognition. Hydrogen bonds lock guanine in place with specificity, while cation–π interactions provide strong electrostatic interaction support, and π–π stacking further stabilizes the binding complex.

- Hydrogen bonds, particularly those involving N2 and O6 atoms of the guanine base, confer specificity to guanine recognition by distinguishing it from adenine.
- Quantum chemical analysis revealed the critical role of cation–π interactions between the guanine ring and its surrounding basic residues (Lys and Arg) in stabilizing guanine binding within GTP-binding proteins. Intermolecular interaction energies for representative cation–π interactions range from −1.51 to −10.61 kcal/mol. The high-energy strength of cation–π interactions can be attributed to the multi-mode intermolecular interactions associated with the Lys and Arg residues. For example, the Lys residue of the NKXD motif can be involved in both the cation–π interactions between the positively charged ε-amino groups of lysine and the guanine ring, as well as in the CH–π interactions between the side chain alkyl groups of lysine and the guanine ring.
- π–π stacking interactions between the guanine ring and its surrounding aromatic residues (Phe, Tyr, and Trp) act as an auxiliary stabilizing factor. In complex featuring the NKXD motif, those aromatic residues are typically situated on the opposite side of the guanine ring relative to the Lys residue of the NKXD motif (see, for example, the table of content figure). Intermolecular interaction energies for representative π–π stacking interactions range from −0.34 to −6.57 kcal/mol.

This combination of non-bonded interaction modes maximizes both the strength and selectivity of the molecular recognition of guanine in GTP-binding proteins.

The collective insights gleaned from these investigations illuminate the sophisticated molecular recognition strategies employed by GTP-binding proteins. By harnessing a combination of hydrogen bonding, cation–π, and π–π stacking interactions, these proteins achieve the requisite specificity and affinity for effective guanine binding. This versatile interaction framework not only stabilizes guanine within diverse protein families, but also underpins the essential biological functions of GTP-binding proteins in various cellular processes.

## Figures and Tables

**Table 1:** List of protein complexes with bound guanine nucleotides.

PDB ID	Protein	Resolution (Å)	Nucleotide ^a^	Family ^b^
5DN8	GTPase Der	1.76	GDP	50S ribosome-binding GTPase
5X4B	GTPase Der	1.50	GDP	50S ribosome-binding GTPase
5M04	GTPase ObgE/CgtA	1.85	GDP	50S ribosome-binding GTPase
2DBY	GTP-binding protein	1.76	GDP	50S ribosome-binding GTPase
2DYK	GTP-binding protein	1.96	GDP	50S ribosome-binding GTPase
4DCU	GTP-binding protein ENGA	2.00	GDP	50S ribosome-binding GTPase
3EC1	GTP-binding protein YqeH	2.36	GDP	50S ribosome-binding GTPase
1SVI	GTP-binding protein YSXC	1.95	GDP	50S ribosome-binding GTPase
1MKY	Probable GTP-binding protein EngA	1.90	GDP	50S ribosome-binding GTPase
3PQC	Probable GTP-binding protein EngB	1.90	GDP	50S ribosome-binding GTPase
5UCV	Probable GTP-binding protein EngB	1.80	GDP	50S ribosome-binding GTPase
3CNO	Putative uncharacterized protein	2.30	GDP	50S ribosome-binding GTPase
3QXX	Dethiobiotin synthetase	1.36	GDP	AAA domain
2FQX	Membrane lipoprotein tmpC	1.70	GMP	ABC transporter substrate-binding protein PnrA-like
4FWP	Propionate kinase	2.50	GDP	Acetokinase family
2QVN	Adenosine deaminase	2.19	GMP	Adenosine deaminase
1LON	Adenylosuccinate synthetase	2.10	GDP	Adenylosuccinate synthetase
1P9B	Adenylosuccinate Synthetase	2.00	GDP	Adenylosuccinate synthetase
1QF5	Adenylosuccinate synthetase	2.00	GDP	Adenylosuccinate synthetase
2V40	Adenylosuccinate synthetase	1.90	GDP	Adenylosuccinate synthetase
5I34	Adenylosuccinate synthetase	1.53	GDP	Adenylosuccinate synthetase
6C25	Adenylosuccinate synthetase	1.90	GDP	Adenylosuccinate synthetase
2X77	ADP-ribosylation factor	2.10	GDP	ADP-ribosylation factor family
1R8S	ADP-ribosylation factor 1	1.46	GDP	ADP-ribosylation factor family
3AQ4	ADP-ribosylation factor 1	1.80	GDP	ADP-ribosylation factor family
3LRP	ADP-ribosylation factor 1	2.50	GDP	ADP-ribosylation factor family
4Y0V	ADP-ribosylation factor 1	1.80	GDP	ADP-ribosylation factor family
2B6H	ADP-ribosylation factor 5	1.76	GDP	ADP-ribosylation factor family
2A5D	ADP-ribosylation factor 6	1.80	GTP	ADP-ribosylation factor family
1UPT	ADP-ribosylation factor-like protein 1	1.70	GTP	ADP-ribosylation factor family
1ZD9	ADP-ribosylation factor-like 10B	1.70	GDP	ADP-ribosylation factor family
1YZG	ADP-ribosylation factor-like 8	2.00	GDP	ADP-ribosylation factor family
2H17	ADP-ribosylation factor-like protein 5A	1.70	GDP	ADP-ribosylation factor family
2H57	ADP-ribosylation factor-like protein 6	2.00	GTP	ADP-ribosylation factor family
1FZQ	ADP-ribosylation factor-like protein3	1.70	GDP	ADP-ribosylation factor family
4V0K	ARF-LIKE SMALL GTPASE	1.438	GDP	ADP-ribosylation factor family
3T1O	Gliding protein mglA	1.90	GDP	ADP-ribosylation factor family
5UF8	Potential ADP-ribosylation factor	1.87	GDP	ADP-ribosylation factor family
3BH7	Protein XRP2	1.90	GDP	ADP-ribosylation factor family
1F6B	SAR1	1.70	GDP	ADP-ribosylation factor family
1H65	CHLOROPLAST OUTER ENVELOPE PROTEIN OEP34	2.00	GDP	AIG1 family
3V70	GTPase IMAP family member 1	2.21	GDP	AIG1 family
2XTM	GTPASE IMAP FAMILY MEMBER 2	1.70	GDP	AIG1 family
3LXX	GTPase IMAP family member 4	2.15	GDP	AIG1 family
3DEF	T7I23.11 protein	1.96	GDP	AIG1 family
4A7W	URIDYLATE KINASE	1.80	GTP	Amino acid kinase family
3ZF8	MANNAN POLYMERASE COMPLEXES SUBUNIT MNN9	1.98	GDP	Anp1
2FP4	Succinyl-CoA ligase [GDP-forming] alpha-chain	2.08	GTP	ATP-grasp domain
3UFX	succinyl-CoA synthetase alpha subunit	2.35	GDP	ATP-grasp domain
4Z87	Inosine-5′-monophosphate dehydrogenase	2.25	GDP	CBS domain
2Q0E	RNA uridylyl transferase	2.10	GTP	Cid1 family poly A polymerase
4LPS	Hydrogenase/urease nickel incorporation protein HypB	2.00	GDP	CobW/HypB/UreG, nucleotide-binding domain
4HI0	Urease accessory protein UreF	2.35	GDP	CobW/HypB/UreG, nucleotide-binding domain
5EY0	GTP-sensing transcriptional pleiotropic repressor CodY	1.60	GTP	CodY GAF-like domain
1YRB	ATP(GTP)binding protein	1.75	GDP	Conserved hypothetical ATP binding protein
5HCI	GPN-loop GTPase 1	2.30	GDP	Conserved hypothetical ATP binding protein
2CXX	Probable GTP-binding protein engB	1.70	GDP	C-terminal region of MMR_HSR1 domain
3LZZ	Putative uncharacterized protein	2.50	GDP	Cupin superfamily (DUF985)
1IH7	DNA primase	2.21	GMP	DNA polymerase family B
4EDK	DNA primase	2.00	GTP	DNA primase catalytic core, N-terminal domain
4B2P	DNA recombination and repair protein Rad51-like	1.60	GTP	DNA recombination/repair protein RadA
5D3Q	Dynamin-1,Dynamin-1	1.70	GDP	Dynamin family
3W6P	Dynamin-1-like protein	1.70	GDP	Dynamin family
3L43	Dynamin-3	2.27	GDP	Dynamin family
4P4T	Interferon-induced GTP-binding protein Mx1	2.30	GDP	Dynamin family
1KK3	eIF2gamma	1.90	GDP	Elongation factor Tu GTP-binding domain
4TMX	eIF5B	1.50	GTP	Elongation factor Tu GTP-binding domain
1IJE	Elongation factor 1-alpha	2.40	GDP	Elongation factor Tu GTP-binding domain
1SKQ	Elongation factor 1-alpha	1.80	GDP	Elongation factor Tu GTP-binding domain
3WXM	Elongation factor 1-alpha	2.30	GTP	Elongation factor Tu GTP-binding domain
3WY9	Elongation factor 1-alpha	2.30	GDP	Elongation factor Tu GTP-binding domain
5H7K	Elongation factor 2	1.60	GDP	Elongation factor Tu GTP-binding domain
2BM0	Elongation factor G	2.40	GDP	Elongation factor Tu GTP-binding domain
2DY1	Elongation factor G	1.60	GTP	Elongation factor Tu GTP-binding domain
1D2E	Elongation factor Tu	1.94	GDP	Elongation factor Tu GTP-binding domain
1HA3	Elongation factor Tu	2.00	GDP	Elongation factor Tu GTP-binding domain
4J0Q	Elongation factor Tu-A	2.30	GDP	Elongation factor Tu GTP-binding domain
4NCN	Eukaryotic translation initiation factor 5B-like protein	1.87	GTP	Elongation factor Tu GTP-binding domain
2YWH	GTP-binding protein LepA	2.24	GDP	Elongation factor Tu GTP-binding domain
3TR5	Peptide chain release factor 3	2.11	GDP	Elongation factor Tu GTP-binding domain
3VQT	Peptide chain release factor 3	1.80	GDP	Elongation factor Tu GTP-binding domain
5FG3	Probable translation initiation factor IF-2	1.90	GDP	Elongation factor Tu GTP-binding domain
2HCJ	Protein chain elongation factor EF-Tu	2.12	GDP	Elongation factor Tu GTP-binding domain
4ZKD	Superkiller protein 7	2.18	GDP	Elongation factor Tu GTP-binding domain
4B47	Translation initiation factor IF-2	2.30	GDP	Elongation factor Tu GTP-binding domain
1G7S	Translation initiation factor IF2/EIF5B	2.00	GDP	Elongation factor Tu GTP-binding domain
4RD1	Translation initiation factor 2 subunit gamma	1.50	GDP	Elongation factor Tu GTP-binding domain
2PHN	F420-0:gamma-glutamyl ligase	1.35	GDP	F420-0:Gamma-glutamyl ligase
3I8X	Ferrous iron transport protein B	2.25	GDP	Ferrous iron transport protein B
3W5J	Ferrous iron transport protein B	1.93	GDP	Ferrous iron transport protein B
2WJH	Ferrous iron transport protein B HOMOLOG	2.10	GDP	Ferrous iron transport protein B
4NON	Ferrous iron uptake transporter protein B	2.50	GDP	Ferrous iron transport protein B
3A1S	Iron(II) transport protein B	1.50	GDP	Ferrous iron transport protein B
4HDG	Polyprotein	2.38	GTP	Flavivirus RNA-directed RNA polymerase
5VYR	Formyltransferase	1.70	GMP	Formyl transferase
2PXA	Genome polyprotein	2.30	GTP	FtsJ-like methyltransferase
4V0R	NS5 POLYMERASE	2.40	GTP	FtsJ-like methyltransferase
3EVD	RNA-directed RNA polymerase NS5	1.50	GTP	FtsJ-like methyltransferase
5GOZ	RNA-directed RNA polymerase NS5	2.05	GTP	FtsJ-like methyltransferase
5U32	tRNA ligase	2.19	GDP	Fungal tRNA ligase phosphodiesterase domain
3ZY2	GDP-fucose protein O-fucosyltransferase 1	1.54	GDP	GDP-fucose protein O-fucosyltransferase
5KXH	GDP-fucose protein O-fucosyltransferase 1	1.33	GDP	GDP-fucose protein O-fucosyltransferase
5FOE	GDP-fucose protein O-fucosyltransferase 2,Thrombospondin-1	1.98	GDP	GDP-fucose protein O-fucosyltransferase
2Z1M	GDP-D-mannose dehydratase	2.00	GDP	GDP-mannose 4,6 dehydratase
1N7H	GDP-D-mannose-4,6-dehydratase	1.80	GDP	GDP-mannose 4,6 dehydratase
5IN4	GDP-mannose 4,6 dehydratase	1.60	GDP	GDP-mannose 4,6 dehydratase
1RPN	GDP-mannose 4,6-dehydratase	2.15	GDP	GDP-mannose 4,6 dehydratase
5UZH	NafoA.00085.b	2.25	GDP	GDP-mannose 4,6 dehydratase
6DHM	Glutamate dehydrogenase 1, mitochondrial	3.00	GTP	Glutamate/Leucine/Phenylalanine/Valine dehydrogenase
1S4O	Glycolipid 2-alpha-mannosyltransferase	2.01	GDP	Glycolipid 2-alpha-mannosyltransferase
5A07	Mannosyltransferase KTR4	1.90	GDP	Glycolipid 2-alpha-mannosyltransferase
5MLZ	Dolichol monophosphate mannose synthase	2.00	GDP	Glycosyl transferase family 2
2Y4K	MANNOSYLGLYCERATE SYNTHASE	2.45	GDP	Glycosyl transferase family 2
3OKC	Mannosyltransferase	2.40	GDP	Glycosyl transferases group 1
4N9W	Mannosyltransferase	1.94	GDP	Glycosyl transferases group 1
2NZX	Alpha1,3-fucosyltransferase	1.90	GDP	Glycosyltransferase family 10 (fucosyltransferase) C-term
4F97	VldE	2.11	GDP	Glycosyltransferase family 20
1ZCB	G alpha i/13	2.00	GDP	G-protein alpha subunit
2ODE	Guanine nucleotide-binding protein G(k) subunit alpha	1.90	GDP	G-protein alpha subunit
1TAD	TRANSDUCIN-ALPHA	1.70	GDP	G-protein alpha subunit
4DU6	GTP cyclohydrolase 1	2.11	GTP	GTP cyclohydrolase I
1A8R	GTP CYCLOHYDROLASE I	2.11	GTP	GTP cyclohydrolase I
2QV6	GTP cyclohydrolase III	2.00	GTP	GTP cyclohydrolase III
2QTH	GTP-binding protein	2.00	GDP	GTP-binding GTPase Middle Region
1ZNY	Guanylate kinase	2.30	GDP	Guanylate kinase
2AN9	Guanylate kinase	2.35	GDP	Guanylate kinase
6B9F	Atlastin-1	1.90	GDP	Guanylate-binding protein, N-terminal domain
5VGR	Atlastin-3	2.10	GDP	Guanylate-binding protein, N-terminal domain
1VJ7	Bifunctional RELA/SPOT	2.10	GDP	HD domain
4TNP	Deoxynucleoside triphosphate triphosphohydrolase SAMHD1	2.00	GTP	HD domain
2HEK	Hypothetical protein	1.99	GDP	HD domain
2OGI	Hypothetical protein SAG1661	1.85	GDP	HD domain
4TZ0	ATP-dependent RNA helicase MSS116, mitochondrial	2.35	GDP	Helicase conserved C-terminal domain
4XBA	Aprataxin-like protein	1.50	GMP	HIT domain
5AQK	HEAT SHOCK COGNATE 71 KDA PROTEIN	2.09	GDP	Hsp70 protein
4Q46	Polymerase basic protein 2	1.80	GDP	Influenza RNA-dependent RNA polymerase subunit PB2
4LV5	Rhoptry protein 5B	1.70	GDP	Interferon-inducible GTPase (IIGP)
5GMF	Toll-like receptor 7	2.50	GMP	Leucine-rich repeat
4NXV	Mitochondrial dynamic protein MID51	2.30	GDP	Mab-21 protein
2ZU9	Mannosyl-3-phosphoglycerate synthase	2.00	GDP	Mannosyl-3-phosphoglycerate synthase (osmo_MPGsynth)
3NXS	LAO/AO transport system ATPase	2.30	GDP	Methylmalonyl Co-A mutase-associated GTPase MeaB
3TK1	Membrane ATPase/protein kinase	2.40	GDP	Methylmalonyl Co-A mutase-associated GTPase MeaB
4LC1	Methylmalonyl-CoA mutase accessory protein	1.80	GDP	Methylmalonyl Co-A mutase-associated GTPase MeaB
3P32	Probable GTPase Rv1496/MT1543	1.90	GDP	Methylmalonyl Co-A mutase-associated GTPase MeaB
3DMH	Probable ribosomal RNA small subunit methyltransferase	1.55	GMP	Methyltransferase small domain
1FRW	MOLYBDOPTERIN-GUANINE DINUCLEOTIDE BIOSYNTHESIS PROTEIN	1.75	GTP	MobA-like NTP transferase domain
2FB3	Molybdenum cofactor biosynthesis protein A	2.35	GTP	Molybdenum Cofactor Synthesis C
1SIW	Respiratory nitrate reductase 1 alpha chain	2.20	GDP	Molybdopterin oxidoreductase
1CKM	MRNA CAPPING ENZYME	2.50	GTP	mRNA capping enzyme, catalytic domain
4PZ6	mRNA-capping enzyme subunit alpha	2.41	GMP	mRNA capping enzyme, catalytic domain
1JWY	Myosin-2 heavy chain, Dynamin-A	2.30	GDP	Myosin head (motor domain)
3SIW	Nodulation fucosyltransferase NodZ	1.98	GDP	Nodulation protein Z (NodZ)
2E87	Hypothetical protein PH1320	2.35	GDP	NOG1 N-terminal helical domain
2DXE	Nucleoside diphosphate kinase	1.70	GDP	Nucleoside diphosphate kinase
3BBF	Nucleoside diphosphate kinase B	1.70	GDP	Nucleoside diphosphate kinase
1RYA	GDP-mannose mannosyl hydrolase	1.30	GDP	NUDIX domain
2A8S	U8 snoRNA-binding protein X29	2.45	GTP	NUDIX domain
4LC4	Probable sugar kinase protein	1.70	GMP	pfkB family carbohydrate kinase
2FAH	Phosphoenolpyruvate carboxykinase	2.09	GDP	Phosphoenolpyruvate carboxykinase C-terminal P-loop domain
4R43	Phosphoenolpyruvate carboxykinase [GTP]	1.80	GDP	Phosphoenolpyruvate carboxykinase C-terminal P-loop domain
1JE1	5′-METHYLTHIOADENOSINE PHOSPHORYLASE	1.80	GMP	Phosphorylase superfamily
1ODJ	PURINE NUCLEOSIDE PHOSPHORYLASE	2.40	GMP	Phosphorylase superfamily
3IEX	Purine-nucleoside phosphorylase	2.05	GMP	Phosphorylase superfamily
4DT9	APH(2″)-Id	2.10	GMP	Phosphotransferase enzyme family
4ORK	Bifunctional AAC/APH	2.30	GDP	Phosphotransferase enzyme family
3TDW	Gentamicin resistance protein	1.70	GDP	Phosphotransferase enzyme family
5IGI	Macrolide 2′-phosphotransferase	1.20	GMP	Phosphotransferase enzyme family
5IH1	Macrolide 2′-phosphotransferase II	1.31	GDP	Phosphotransferase enzyme family
5UXC	Predicted aminoglycoside phosphotransferase	1.72	GDP	Phosphotransferase enzyme family
3LDU	Putative methylase	1.70	GTP	Putative RNA methylase family UPF0020
5JCP	Arf-GAP with Rho-GAP domain	2.10	GDP	Ras family
5OEC	GtgE	2.30	GDP	Ras family
2G77	GTPase-activating protein GYP1	2.26	GDP	Ras family
4DJT	GTP-binding nuclear protein GSP1	1.80	GDP	Ras family
3M1I	GTP-binding nuclear protein GSP1/CNR1	2.00	GTP	Ras family
3GJ0	GTP-binding nuclear protein Ran	1.48	GDP	Ras family
2GF0	GTP-binding protein Di-Ras1	1.90	GDP	Ras family
2ERX	GTP-binding protein Di-Ras2	1.65	GDP	Ras family
2G3Y	GTP-binding protein GEM	2.40	GDP	Ras family
2DPX	GTP-binding protein RAD	1.80	GDP	Ras family
2NZJ	GTP-binding protein REM 1	2.50	GDP	Ras family
3CBQ	GTP-binding protein REM 2	1.82	GDP	Ras family
6BSX	GTP-binding protein Rheb	1.65	GDP	Ras family
4KLZ	GTP-binding protein Rit1	2.30	GDP	Ras family
3RWO	GTP-binding protein YPT32/YPT11	1.70	GDP	Ras family
1KY3	GTP-BINDING PROTEIN YPT7P	1.35	GDP	Ras family
2ZEJ	Leucine-rich repeat kinase 2	2.00	GDP	Ras family
5UB8	Likely Rab family GTP-binding protein	2.35	GDP	Ras family
2WKQ	NPH1-1, RAS-RELATED C3 BOTULINUM TOXIN SUBSTRATE 1	1.60	GTP	Ras family
1QRA	P21RAS	1.60	GTP	Ras family
5XC5	Probable Rab-related GTPase	1.40	GTP	Ras family
1EK0	PROTEIN (GTP-BINDING PROTEIN YPT51)	1.48	GDP	Ras family
2F9L	RAB11B, member RAS oncogene family	1.55	GDP	Ras family
2IL1	Rab12	2.10	GDP	Ras family
1Z0F	RAB14, member RAS oncogene family	2.15	GDP	Ras family
3CLV	Rab5 protein, putative	1.89	GDP	Ras family
1D5C	RAB6 GTPASE	2.30	GDP	Ras family
3BWD	Rac-like GTP-binding protein ARAC6	1.53	GDP	Ras family
2J0V	RAC-LIKE GTP-BINDING PROTEIN ARAC7	1.78	GDP	Ras family
1KAO	RAP2A	1.70	GDP	Ras family
2P5S	RAS and EF-hand domain containing	2.15	GDP	Ras family
2Q3H	Ras homolog gene family, member U	1.73	GDP	Ras family
5WDS	Ras protein	1.85	GDP	Ras family
2ATV	RAS-like estrogen-regulated growth inhibitor	1.90	GDP	Ras family
3C5C	RAS-like protein 12	1.85	GDP	Ras family
5O33	Ras-related C3 botulinum toxin substrate 1	1.64	GDP	Ras family
5VCU	Ras-related c3 botulinum toxin substrate 1 isoform x2	1.85	GDP	Ras family
3KKQ	Ras-related protein M-Ras	1.20	GDP	Ras family
1Z0I	Ras-related protein Rab-21	2.33	GDP	Ras family
1Z0J	Ras-related protein Rab-22A	1.32	GTP	Ras family
1Z2A	Ras-related protein Rab-23	1.90	GDP	Ras family
2OIL	Ras-related protein Rab-25	2.30	GDP	Ras family
1Z0A	Ras-related protein Rab-2A	2.12	GDP	Ras family
2A5J	Ras-related protein Rab-2B	1.501	GDP	Ras family
3DZ8	Ras-related protein Rab-3B	1.90	GDP	Ras family
2GF9	Ras-related protein Rab-3D	1.53	GDP	Ras family
2HUP	RAS-related protein RAB-43	2.05	GDP	Ras family
2BMD	RAS-RELATED PROTEIN RAB4A	1.80	GDP	Ras family
2O52	Ras-related protein Rab-4B	2.20	GDP	Ras family
1N6K	Ras-related protein Rab-5A	1.55	GDP	Ras family
2E9S	Ras-related protein Rab-6B	1.78	GDP	Ras family
1T91	Ras-related protein Rab-7	1.90	GTP	Ras family
4LHV	Ras-related protein Rab-8A	1.95	GDP	Ras family
1WMS	Ras-related protein Rab-9A	1.25	GDP	Ras family
4QXA	Ras-related protein Rab-9A	2.30	GTP	Ras family
1U8Z	Ras-related protein Ral-A	1.50	GDP	Ras family
3X1W	Ras-related protein Rap-1b	1.20	GDP	Ras family
2FN4	Ras-related protein R-Ras	1.65	GDP	Ras family
2ERY	Ras-related protein R-Ras2	1.70	GDP	Ras family
4MIT	Rho family GTPase	2.35	GTP	Ras family
3REF	Rho-like small GTPase	1.95	GDP	Ras family
2CLS	Rho-related GTP-binding protein RHO6	2.31	GTP	Ras family
2FV8	Rho-related GTP-binding protein RhoB	1.90	GDP	Ras family
2J1L	Rho-related GTP-binding protein RHOD	2.50	GDP	Ras family
1M7B	Rnd3/RhoE small GTP-binding protein	2.00	GTP	Ras family
2BCG	Secretory pathway GDP dissociation inhibitor	1.48	GDP	Ras family
2EFH	Similarity to vacuolar protein sorting-associated protein VPS9	2.10	GDP	Ras family
3BFK	Small GTPase Rab11	1.80	GDP	Ras family
5C4M	Transforming protein RhoA	1.30	GDP	Ras family
5C2K	Transforming protein RhoA, Rac GTPase-activating protein 1	1.42	GDP	Ras family
6EWZ	GTP pyrophosphokinase	2.24	GTP	Region found in RelA/SpoT proteins
3O0Q	Ribonucleoside-diphosphate reductase	1.80	GDP	Ribonucleotide reductase, all-alpha domain
2CVW	Ribonucleoside-diphosphate reductase large chain 1	2.40	GDP	Ribonucleotide reductase, barrel domain
5CA8	Protein SEY1	2.30	GDP	Root hair defective 3 GTP-binding protein (RHD3)
2RCN	Probable GTPase EngC	2.25	GDP	RsgA GTPase
2YV5	YjeQ protein	1.90	GDP	RsgA GTPase
4Z54	Neuronal-specific septin-3	1.83	GDP	Septin
4KV9	Septin	1.93	GDP	Septin
5CYO	Septin-9	2.04	GDP	Septin
2FH5	Signal recognition particle receptor alpha subunit	2.45	GTP	Signal recognition particle receptor beta subunit
1NRJ	Signal recognition particle receptor alpha subunit homolog	1.70	GTP	Signal recognition particle receptor beta subunit
2IYL	Cell division protein FTSY	2.10	GDP	SRP54-type protein, GTPase domain
5L3V	Signal recognition particle 54 kDa protein	2.30	GDP	SRP54-type protein, GTPase domain
2C03	Signal recognition particle receptor	1.24	GDP	SRP54-type protein, GTPase domain
3E70	Signal recognition particle receptor	1.97	GDP	SRP54-type protein, GTPase domain
5L3W	Signal recognition particle receptor FtsY	2.40	GDP	SRP54-type protein, GTPase domain
2ZGY	Plasmid segregation protein parM	1.90	GDP	StbA protein
4IEN	Putative acyl-CoA hydrolase	2.00	GDP	Thioesterase superfamily
1OFU	Cell division protein FtsZ	2.10	GDP	Tubulin/FtsZ family, GTPase domain
2R6R	Cell division protein ftsZ	1.70	GDP	Tubulin/FtsZ family, GTPase domain
2RHL	Cell Division Protein ftsZ	2.45	GDP	Tubulin/FtsZ family, GTPase domain
2VAP	Cell division protein FtsZ	1.70	GDP	Tubulin/FtsZ family, GTPase domain
4B46	Cell division protein ftsZ	1.90	GDP	Tubulin/FtsZ family, GTPase domain
5XDT	Cell division protein FtsZ	1.30	GDP	Tubulin/FtsZ family, GTPase domain
4EI7	Plasmid replication protein RepX	1.90	GDP	Tubulin/FtsZ family, GTPase domain
5IYZ	Tubulin alpha-1B chain	1.80	GTP	Tubulin/FtsZ family, GTPase domain
2BTO	TUBULIN BTUBA	2.50	GTP	Tubulin/FtsZ family, GTPase domain
3CB2	Tubulin gamma-1 chain	2.30	GDP	Tubulin/FtsZ family, GTPase domain
3ZID	TUBULIN/FTSZ, GTPASE	2.00	GDP	Tubulin/FtsZ family, GTPase domain
4XCQ	TubZ	2.39	GDP	Tubulin/FtsZ family, GTPase domain
1RA7	Genome polyprotein	2.35	GTP	Viral RNA-dependent RNA polymerase
1UVK	RNA-dependent RNA polymerase	2.45	GTP	Viral RNA-dependent RNA polymerase
5XE0	Genome polyprotein	2.30	GTP	Viral RNA-dependent RNA polymerase
3N6M	RNA-dependent RNA polymerase	2.50	GTP	Viral RNA-dependent RNA polymerase
4UCI	RNA-dependent RNA polymerase L	2.21	GTP	Virus-capping methyltransferase
5KWK	Galactoside 2-alpha-L-fucosyltransferase	1.90	GDP	Xyloglucan fucosyltransferase
2GJ8	tRNA modification GTPase trmE	1.70	GDP	50S ribosome-binding GTPase
2IRX	DNA ligase-like protein Rv0938/MT0965	1.80	GTP	DNA primase small subunit
5KSP	Mitochondrial Rho GTPase 1	2.16	GDP	Ras family
5KU1	Mitochondrial Rho GTPase 1	2.50	GDP	Ras family
5KUT	Mitochondrial Rho GTPase 2	1.69	GDP	Ras family
3ZBQ	PHIKZ039	1.70	GDP	Tubulin/FtsZ family, GTPase domain
3R4V	Putative uncharacterized protein	1.67	GDP	Tubulin/FtsZ family, GTPase domain
3DZH	ADP-ribosyl cyclase 1	1.60	GTP	-
4XJ3	Cyclic AMP-GMP synthase	1.65	GTP	-
3T34	Dynamin-related protein 1AA	2.41	GDP	-
1MRE	IGG2B-KAPPA JEL103 FAB (LIGHT CHAIN)	2.30	GDP	-
4XUL	mg662	2.26	GTP	-
5GOF	Mitofusin-1	1.60	GTP	-
5X6Z	mRNA capping enzyme P5	2.10	GDP	-
4GMU	Phosphoenolpyruvate carboxykinase	1.20	GTP	-
3WNC	Protein translation elongation factor 1A	1.90	GDP	-
2QU8	Putative nucleolar GTP-binding protein 1	2.01	GDP	-
5CK4	Putative signal recognition particle protein	1.89	GDP	-
4KU4	Ras-3 from Cryphonectria parasitica	1.60	GDP	-
3SFV	Ras-related protein Rab-1A	1.73	GDP	-
2RHD	Small GTP-binding protein rab1a	2.06	GDP	-
1JLR	Uracil Phosphoribosyltransferase	2.45	GTP	-

^a^ GTP, guanosine-5′-triphosphate; GDP, guanosine-5′-diphosphate; and GMP, guanosine-5′-monophosphate. ^b^ Family of proteins according to the Pfam classification [34]. Dashed line “-“ indicates that no knowledge of Pfam classification for the protein is available.

**Table 2:** Summary of the hydrogen bond patterns between guanine and the surrounding residues and their associated sequence motifs.

S. N.	Motif	Hydrogen Bonding Pattern ^a^	Percentage/No. of Complexes
1.	NKXD	**N_i_-K_i+1_**-X_i+2_-**D_i+3_**	19.1%: 57 out of 298
2.	NKXD	**N_i_**-K_i+1_-X_i+2_-**D_i+3_**	19.1%: 57 out of 298
3.	NKXD	N_i_-**K_i+1_**-X_i+2_-**D_i+3_**	6.7%: 20 out of 298
4.	NKXD	N_i_-K_i+1_-X_i+2_-**D_i+3_**	11.7%: 35 out of 298
5.	Non-NKXD	D_i_/E_i_ plus	20.5%: 61 out of 298
6.	Non-NKXD	Others	22.8%: 68 out of 298

^a^. The uppercase letters represent the NKXD motif, and “i” in the subscript represents the residue number. The amino acid that forms the hydrogen bond with the guanine ring is colored in red text with a bold face. The cut-off distance for hydrogen bonds is 3.5 Å.

**Table 3:** Pairwise interaction energies for representative intermolecular cation–π interacting pairs.

S. N	Cation–π Interaction Pair ^a^	PDB ID	Distance ^b^ (Å)	${\Delta E}_{Int}^{gas}$ (Kcal/mol) ^c^	${\Delta E}_{Deh}$ (Kcal/mol)	${\Delta E}_{Int}^{aq}$ ^d^ (Kcal/mol)
1.	G…K131	1G7S	3.44	−10.74	0.13	−10.61
2.	G…K124	2BMD	4.25	−6.07	−4.23	−10.30
3.	G…R130	1S4O	3.42	−8.51	−1.30	−9.81
4.	G…K205	3P32	4.26	−6.49	−2.89	−9.38
5.	G…R52	1RYA	2.77	−33.65	24.57	−9.08
6.	G…K126	1T91	3.87	−4.05	−4.76	−8.81
7.	G…K55	2IRX	2.97	−36.38	28.30	−8.09
8.	G…R142	5A07	3.57	−6.38	−1.56	−7.94
9.	G…R217	6B9F	2.87	−28.58	23.07	−5.51
10.	G…R181	4B2P	3.73	−9.12	4.59	−4.53
11.	G…R90	2DYK	4.29	−2.78	0.72	−2.06
12.	G…K45	2V40	4.92	−11.4	9.89	−1.51

^a^ “G” represents guanine. ^b^ The intermolecular distance between the positively charged residue and the guanine base. ^c^ Gas-phase interaction energies calculated at the B2PLYP-D3/cc-pVDZ level of theory. ^d^ Solution-phase interaction energies were calculated according to the equation ${\Delta E}_{Int}^{aq}$ = ${\Delta E}_{Int}^{gas}$ + ${\Delta E}_{Deh}$, as described in the Theory and Methods section.

**Table 4:** Pairwise interaction energies for representative π–π stacking interaction pairs.

S. N	π–π Stacking Pair ^a^	PDB ID	Angle (Degrees)	Distance ^b^ (Å)	${\Delta E}_{Int}^{gas}$ (Kcal/mol) ^c^	${\Delta E}_{Deh}$ (Kcal/mol)	${\Delta E}_{Int}^{aq}$ (Kcal/mol) ^d^
1.	G…F3	1RYA	18.69	3.29	−4.58	−1.99	−6.57
2.	G…Y161	3R4V	10.94	3.53	−5.36	−1.03	−6.39
3.	G…W189	3DZH	2.95	3.40	−7.13	1.08	−6.05
4.	G…Y630	1UVK	8.39	3.46	−3.59	−2.38	−5.97
5.	G…F24	3EVD	8.70	3.30	−4.38	−0.99	−5.37
6.	G…Y94	5IGI	20.39	3.51	−9.69	6.14	−3.55
7.	G…W359	4Q46	9.77	3.40	−4.13	0.61	−3.52
8.	G…F160	1JE1	53.83	3.54	−3.06	−0.18	−3.24
9.	G…F28	3KKQ	77.41	3.98	−2.4	0.84	−1.56
10.	G…F227	5VYR	56.85	4.04	−2.75	1.73	−1.02
11.	G…F293	6B9F	78.43	3.96	−1.07	0.26	−0.81
12.	G…F277	1RPN	15.53	3.46	−0.67	0.04	−0.63
13.	G…Y344	4XUL	12.69	5.28	−3.29	2.92	−0.37
14.	G…F190	4LPS	46.47	3.48	−1.68	1.34	−0.34

^a^ “G” represents guanine. ^b^ The intermolecular distance between the aromatic residue and the guanine base. ^c^ Gas-phase interaction energies calculated at the B2PLYP-D3/cc-pVDZ level of theory. ^d^ Solution-phase interaction energies calculated according to the equation ${\Delta E}_{Int}^{aq}$ = ${\Delta E}_{Int}^{gas}$ + ${\Delta E}_{Deh}$, as described in the Theory and Methods section.

**Table 5:** The interaction energies for various modes of non-bonded interactions between the guanine base and its surrounding residues in p21-ras (PDB ID: 1QRA).

S. N.	Intermolecular Pair ^a^	Interaction Mode ^b^	${\Delta E}_{Int}^{gas}$ (Kcal/mol) ^c^	${\Delta E}_{Deh}$ (Kcal/mol)	${\Delta E}_{Int}^{aq}$ (Kcal/mol) ^d^
1.	G…N116	HB	−2.47	1.98	−0.49
2.	G…K117^m^	HB	−6.20	3.62	−2.58
3.	G…D119	HB	−28.59	24.51	−4.08
4.	G…A146^m^	HB	−8.93	6.74	−2.19
5.	G…K117	Cation–π	−5.66	−5.15	−10.81
6.	G…K147	Cation–π	−4.43	−2.96	−7.39
7.	G…F28	π–π	−4.43	1.44	−2.99

^a^ “G” represents guanine; the superscript “m” designates the main chain of the residue. ^b^ HB stands for hydrogen bond interaction. ^c^ The gas-phase interaction energies were calculated at the B2PLYP-D3/cc-pVDZ level of theory. ^d^ The solution-phase interaction energies were calculated according to the equation ${\Delta E}_{Int}^{aq}$ = ${\Delta E}_{Int}^{gas}$ + ${\Delta E}_{Deh}$, as described in the Theory and Methods section.

## Data Availability

All the relevant data are provided in Table 1 of the main text and Table S1 of the Supplementary Materials.

## Supplementary Materials

The following supporting information can be downloaded at: https://www.mdpi.com/article/10.3390/ijms252212449/s1.
